# Preparation and Characterization of Glucose-Based Self-Blowing Non-Isocyanate Polyurethane (NIPU) Foams with Different Acid Catalysts

**DOI:** 10.3390/polym16202899

**Published:** 2024-10-15

**Authors:** Tianjiao Yang, Antonio Pizzi, Xuedong Xi, Xiaojian Zhou, Qianyu Zhang

**Affiliations:** 1 Yunnan Key Laboratory of Wood Adhesives and Glued Products, College of Material Science and Chemistry Engineering, Southwest Forestry University, Kunming 650224, China; 15288047261@163.com (T.Y.); xuedong.xi@swfu.edu.cn (X.X.); xiaojianzhou@hotmail.com (X.Z.); 2 LERMAB, University of Lorraine, 27 Rue Philippe Seguin, 88000 Epinal, France

**Keywords:** glucose-based NIPU, self-blowing, rigid foams, fire resistance, acid catalysts

## Abstract

The preparation and application of non-isocyanate polyurethane (NIPU) from biomass raw materials as a substitute for traditional polyurethane (PU) has recently become a research hot topic as it avoids the toxicity and moisture sensitivity of isocyanate-based PU. In the work presented here, self-blowing GNIPU non-isocyanate polyurethane (NIPU) rigid foams were prepared at room temperature, based on glucose, with acids as catalysts and glutaraldehyde as a cross-linker. The effects of different acids and glutaraldehyde addition on foam morphology and properties were investigated. The water absorption, compressive resistance, fire resistance, and limiting oxygen index (LOI) were tested to evaluate the relevant properties of the foams, and scanning electron microscopy (SEM) was used to observe the foams’ cell structure. The results show that all these foams have a similar apparent density, while their 24 h water absorption is different. The foam prepared with phosphoric acid as a catalyst presented a better compressive strength compared to the other types prepared with different catalysts when above 65% compression. It also presents the best fire resistance with an LOI value of 24.3% (great than 22%), indicating that it possesses a good level of flame retardancy. Thermogravimetric analysis also showed that phosphoric acid catalysis slightly improved the GNIPU foams’ thermal stability. This is mainly due to the flame-retardant effect of the phosphate ion. In addition, scanning electron microscopy (SEM) results showed that all the GNIPU foams exhibited similar open-cell morphologies with the cell pore sizes mainly distributed in the 200–250 μm range.

## 1. Introduction

Polyurethane (PU) foams are prepared by the polymerization foaming of isocyanates and hydroxyl compounds. They have a wide utilization in walls, pipe insulation materials, and building sound insulation materials due to their excellent elasticity, flexibility, elongation, and compressive strength [1,2,3,4]. However, PU foams inevitably require the use of a toxic isocyanate during their preparation and thus are harmful to humans and the environment [5,6]. Therefore, trying to improve their preparation and performance is at present of topical interest.

Currently, biomass foams have become a research hotspot with the development of biomass materials [7,8,9]. Although many works have reported the use of biomass polyols to prepare biomass-based PU foams, the process still uses isocyanates [10,11,12,13,14,15]. Therefore, NIPU has been synthesized to eliminate the harm of isocyanates while satisfying the performance required for polyurethanes [16]. NIPU has safer and more stable characteristics than isocyanate-based PU, while NIPU can present better water absorption, chemical resistance, and penetration resistance. This renders replacing traditional PU feasible, and works have shown that it can be used in coatings, crack-resistant composites, adhesives, and sealants [17,18,19,20,21].

In general, NIPUs were initially prepared by reacting a cyclic carbonate with a polyamine by stepwise addition polymerization. Unfortunately, this method has some drawbacks, such as high pressure [22,23], high temperature [24], catalysts [24], the rather too slow opening of the cyclic carbonate for the further reaction for adhesives, and certain defects in the technology of synthesizing the cyclic carbonate [25,26,27]. Another way has been reported to prepare NIPU: cyclic carbonates were obtained with dimethyl carbonate and polyhydroxyles, and this method has no catalyst and a low time cost [28,29,30,31].

More recently, tannin-based NIPU foams were successfully prepared [32,33,34,35]. Moreover, works on preparing NIPU based on monosaccharides and disaccharides exhibited a good performance, especially applied to wood adhesive [16,30]. The preparation of glucose-based NIPU foams also has been reported [25,26]. However, a drawback is their requirement of a 200 °C temperature for their setting and their poor fire resistance. Their fire resistance has been greatly improved by preparing NIPU foams starting from a mixture of saccharides and tannins [6]. The work presented here is focused on the preparation of rigid foams by adding acid catalysts and aldehyde hardeners for room-temperature preparation and on studying the effect of different acids as catalysts on GNIPU self-blowing rigid foams.

## 2. Materials and Methods

### 2.1. Materials

Glucose (AR), phosphoric acid (AR), and formic acid (AR) were obtained from Guangdong Guanghua Technology Co., Ltd. (Shantou, China); maleic acid (AR) and hexamethylenediamine (AR) were obtained from Sinopharm Chemical Reagent Co., Ltd. (Beijing, China); dimethyl carbonate (AR, 99%) and glutaraldehyde (AR, 50% water solution) were purchased from Shanghai Macklin Biochemical Technology Co., Ltd. (Shanghai, China); citric acid (AR) was obtained from Tianjin Fengchuan Chemical Reagent Technology Co., Ltd. (Tianjin, China).

### 2.2. Synthesis of the Glucose-Based Non-Isocyanate Polyurethane (GNIPU) [25]

In total, 90 g of distilled water, 120 g of glucose, and 108 g of dimethyl carbonate were mixed in a three-necked flask, and the temperature was increased to 70 °C, keeping the reaction for 2 h. After that, 104.4 g of hexamethylene diamine was added to the mixture and stirred; then, the temperature was raised to 90 °C for a 30 min reaction. Next, the resin was cooled to room temperature and discharged.

### 2.3. Preparation of GNIPU Foams

The GNIPU was mixed with an acid (formic acid, maleic acid, phosphoric acid, and citric acid) and glutaraldehyde for foaming at room temperature. The obtained foams were maintained at room temperature for 30 min; then, they were dried in an oven at 73 °C for 10 h.

### 2.4. Water Absorption (24 h) Test

To investigate the effect of different initiators on the water absorption of GNIPU foams, the samples were cut to a size of 2 cm × 2 cm × 2 cm for 24 h water absorption tests. First, the foam was dried in the oven to record the mass (*m*); then, it was completely immersed in water and taken out according to the specified time; the surface moisture was wiped, and then, it was weighed (m′). The calculation formula of water absorption is $\frac{m^{\prime }-m}{m}\times 100\%$. Each sample was tested three times to find the average value.

### 2.5. Compression Test

The obtained GNIPU foams were cut into 2 cm × 2 cm × 2 cm samples and subjected to compression testing. The test was performed on a UTM5105 series electronic universal testing machine (Shenzhen Sansi Zongheng Technology Co., Ltd., Shenzhen, China) in an ambient environment with a testing rate of 2 mm/min.

### 2.6. Ignition Test

Foam samples of 2 cm × 2 cm × 2 cm cut from the foams prepared were placed in the outer flame of an alcohol lamp until the sample charred and did not burn anymore, and the time to reach this state was measured.

### 2.7. Limiting Oxygen Index (LOI)

The limiting oxygen index (LOI) was measured using an FTT0077 oxygen index meter (GBH International, St. Albans, Great Britain). All samples were prepared to a specification size of 80 mm × 10 mm × 10 mm.

### 2.8. TG Analysis

The thermal stability of the foams was studied using TG 209 F3 Tarsus^®^ (Netzsch, Selb, Germany). In total, 5–8 mg of dried powder was placed into a platinum dish, and the sample was heated to the desired temperature at a heating rate of 10 K/min in a nitrogen atmosphere in the temperature range of 30–800 °C.

### 2.9. Fourier Transform Infrared (FT-IR) Spectrometry

To confirm the presence of relevant structures, Fourier transform infrared (FT-IR) analysis was carried out using an iS50 infrared spectrometer (Nicolet, Minneapolis, MN, USA). A blank sample tablet of potassium bromide was prepared for the reference spectrum. A similar tablet was prepared by mixing potassium bromide with 5% *w*/*w* of the sample powders to be analyzed. The spectrum was obtained in transmission measurement by combining 32 scans with a resolution of 2.0 cm^−1^ in the 400–4000 cm^−1^ range.

### 2.10. Scanning Electron Microscopy (SEM)

To investigate the cell microstructure and morphology of foams prepared with different initiators, scanning electron microscopy VEGA3, (TESCAN China, Chengdu, China) was used with an acceleration voltage of 20 kV. The cell size distributions of all foams were calculated by averaging several tens of individual cells for each sample.

## 3. Results and Discussion

### 3.1. GNIPU Foam Reaction Mechanism Analysis

**Figure 1:**
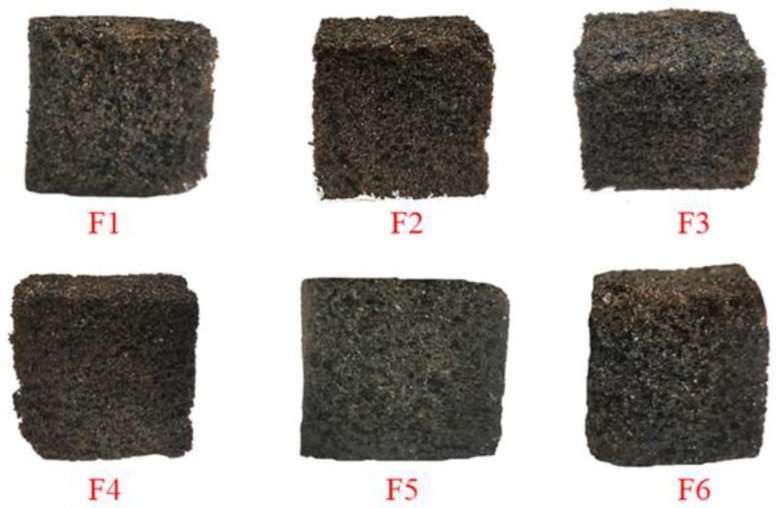
Photograph of GNIPU foams.

**Figure 2:**
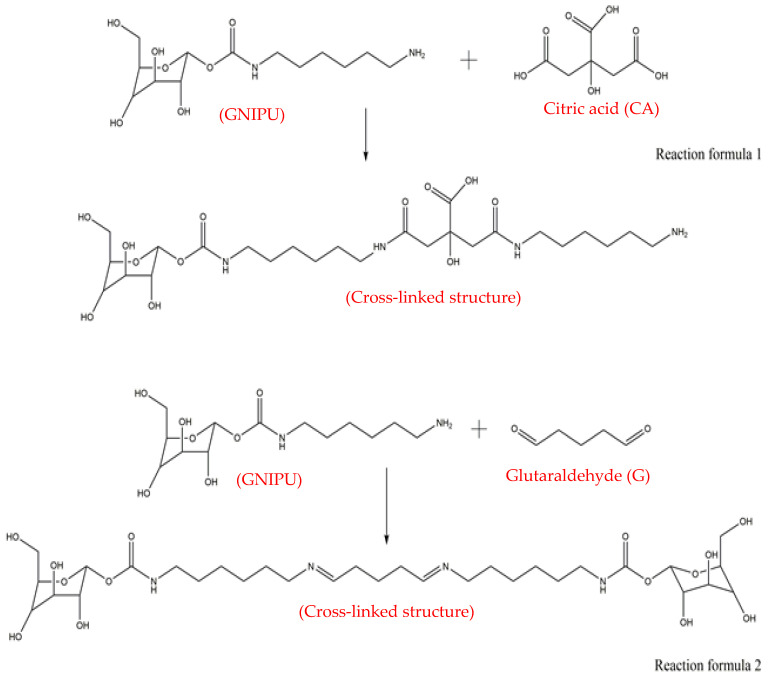
The main reactions leading to a cross-linked network in the foams.

The GNIPU self-blowing foams (F1, F2, F3, F4, F5, and F6) were prepared by adding acids and aldehydes at an ambient temperature. The foam formulations are shown in Table 1, and the samples are shown in Figure 1. From its appearance, the foam prepared with formic acid as an initiator has large cells, and the appearance of the other foams is not much different. The main predictable prepolymers formed by multiple reactions eventually lead to the structural network in the foam, as shown in Figure 2. The whole foaming process can be divided into two main steps: First is the start of the initial formation of the network. Glutaraldehyde is used as a cross-linker and reacts with the amino groups on the glucose-based NIPU prepolymer to ensure that the foam stays in shape (reaction formula (2)). The second step is the foaming process itself. In this stage, the acid reacts with the amino groups to catalyze self-blowing at room temperature (reaction formula (1)). Actually, these two processes occur almost simultaneously leading to finished foam.

### 3.2. Effects of Different Acid and Glutaraldehyde Additions on Basic Foam Properties

Regarding the performance of the GNIPU foams prepared with different catalysts, some of their tested results are shown in Table 1. By comparing foams F1, F2, F3, and F4, it appears that different catalysts do not seem to yield large differences in foam density and ignition time. The formic-acid-catalyzed foam density is only slightly higher than the others. The most likely explanation for this is a lesser catalyst acidity. Formic acid has the weakest acidity of the four acids tried; it reacts relatively gently with amino groups and thus cannot sufficiently catalyze the reaction. This means that mixtures of the same mass can only reach a smaller volume after foaming, thus presenting a higher density. By comparing foams F3, F5, and F6, it can be seen that the density and ignition time of the foams are not much affected by the amount of glutaraldehyde added.

### 3.3. Water Absorption of GNIPU Foams

**Figure 3:**
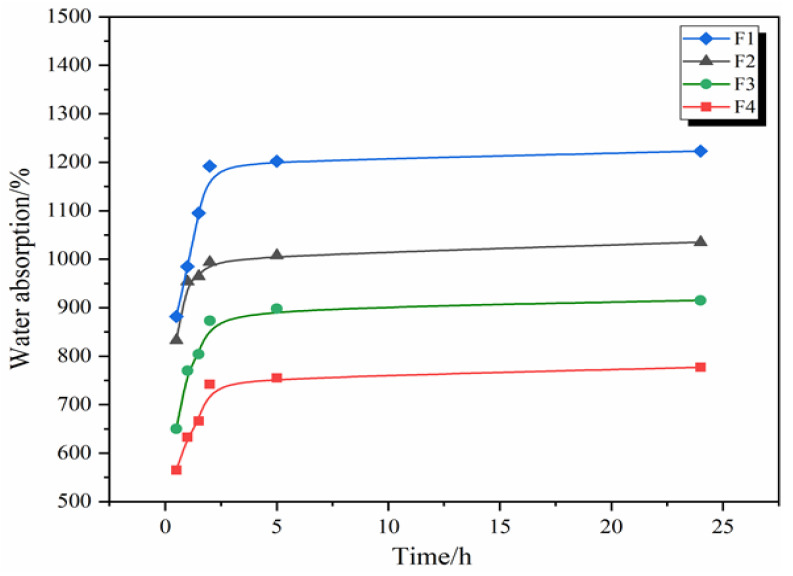
Water absorption (24 h) of GNIPU foams (F1, F2, F3, and F4).

**Figure 4:**
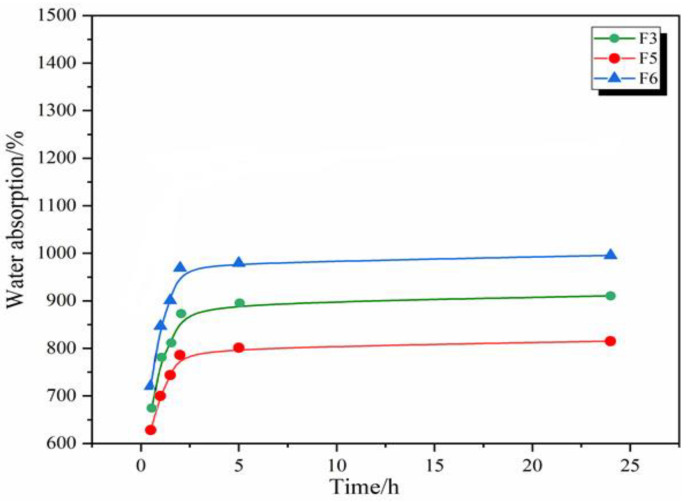
Water absorption (24 h) of GNIPU foams (F3, F5, and F6).

Figure 3 and Figure 4 show the 24 h water absorption of the GNIPU foams prepared according to the formulations in Table 1. First, the GNIPU foams in Figure 3 show different levels of water absorption, this being directly associated with their density and cell opening size. The foams showed a higher water absorption in the first 2.5 h. The water absorption tends to stabilize after about 2.5 h of water immersion due to a certain amount of water already being stored in the cell cavity and thus functioning as reverse hydrostatic pressure. The water absorption appears to be related to foam density, as the curves for lower density foams (F2, F3, and F4) grow slowly, while the denser foams (F1) increase rapidly. Figure 4 shows the same water absorption pattern, where the change in glutaraldehyde addition leads to a different water absorption rate. An increase in the amount of glutaraldehyde led to an improvement in the foam density and thus to a higher water absorption, which is consistent with the conclusion obtained from Figure 3.

### 3.4. Compression Properties of GNIPU Foams

**Figure 5:**
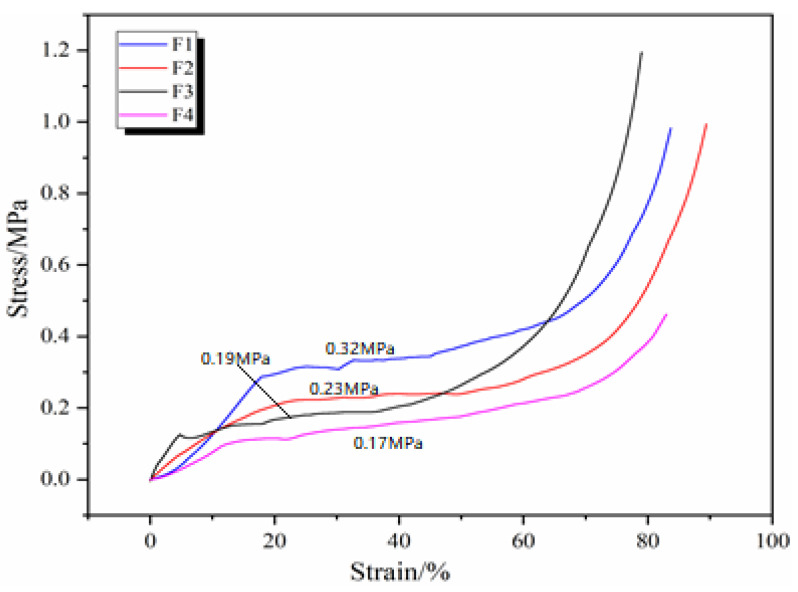
Effects of different initiators on compression performance of GNIPU foams.

Figure 5 shows the compressive stress–strain curves of GNIPU foams F1, F2, F3, and F4 prepared with different acid catalysts. The stress of foam F1 is better than that of the other foams in the compression ratio of 0–65% range, this being due to its much higher density. The stress–strain curves of F2, F3, and F4 are similar in the 0–50% compression ratio range, due to their densities being similar. The reason for this is that the compression resistance is in direct relation to foam density [36]. Theoretically, fragile cell walls can only provide a limited contribution to compression resistance. The foam F1 was observed by scanning electron microscopy (SEM) to be more crumbly than the others, meaning that its cell walls are more brittle. Thus, the cell walls of foam F1 are more easily crushed at a high compression rate, so its stress–strain curves after a compression rate of 65% show a slower rate, with foam F3 showing a better compression resistance instead.

**Figure 6:**
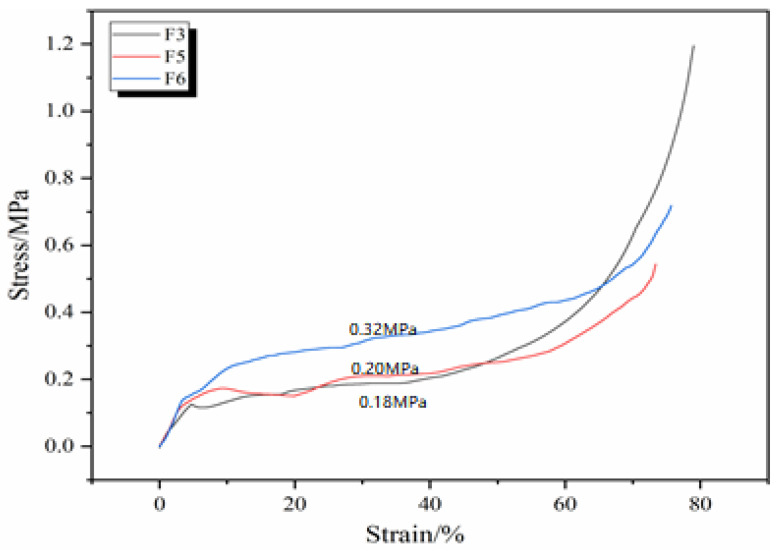
Effect of different additions of glutaraldehyde on compression properties of GNIPU foams.

Figure 6 shows the compressive stress–strain curves of GNIPU foams F3, F5, and F6 prepared with different additions of glutaraldehyde. It can be seen that at the same compression ratio, the more glutaraldehyde is added, the better the compressive properties are. This is because the increase in the proportion of glutaraldehyde yields a more chemically cross-linked network, thus resulting in an improved resistance to compression.

### 3.5. Ignition Test Analysis

**Figure 7:**
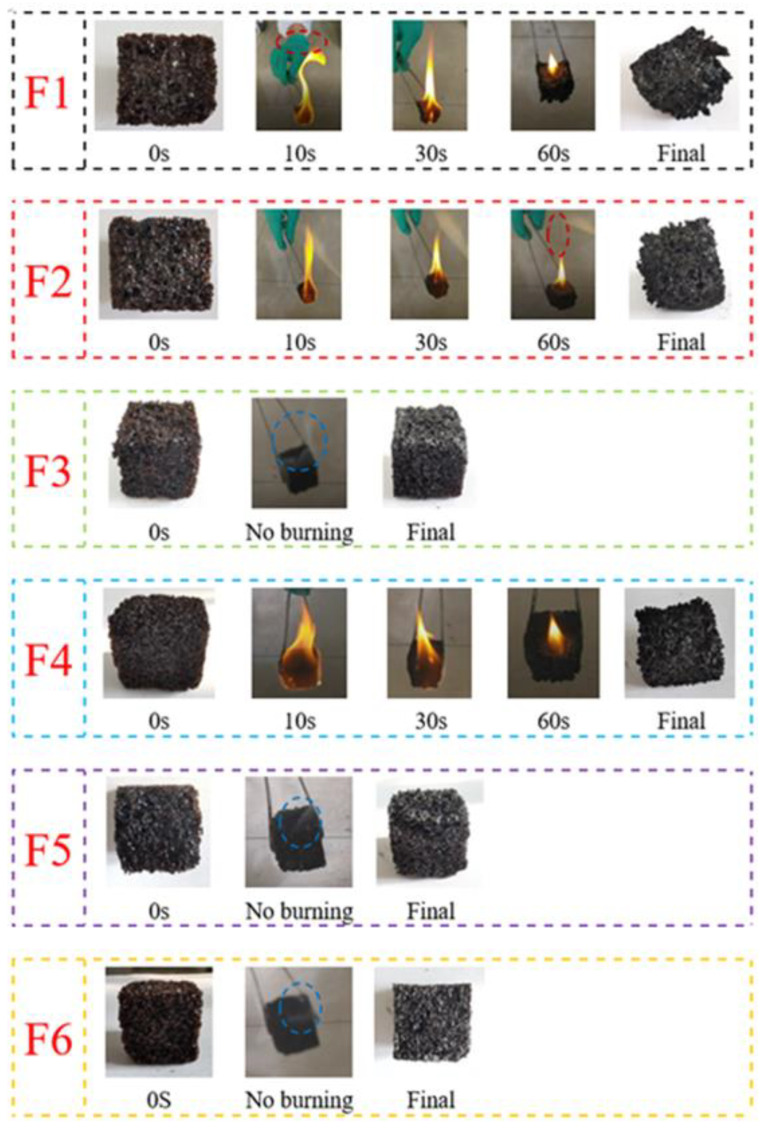
Photographic record of ignition experiments.

The ignition test results of the GNIPU foams are shown in Figure 7. The ignition test indicates to a certain extent the combustion resistance of the material, although with some clear limitations. Nonetheless, the fire resistance of the GNIPU foams prepared with different acid catalysts presents a clear certainty. The foams F1, F2, and F4 were flammable, and a very bright flame was observed in a very short time (10 s). At the same time, a large amount of black fumes (dotted red frame) were released when these foams were burned. Moreover, only completely irregular residues remained after foams F1, F2, and F4 were burned. This shows that these three foams are highly flammable, similarly to isocyanate-based PUs. However, the foams F3, F5, and F6 looked unburned. Furthermore, during ignition, only white smoke appeared, due to water vapor emission, and no large amount of black smoke was released (dotted blue frame), and the samples of foams F3, F5, and F6 after combustion kept their original shape. These results indicate that foam flammability is significantly reduced when the foam is prepared with phosphoric acid as a catalyst. It is well known that phosphorus compounds have been used as flame retardants for traditional isocyanate-based polyurethane foams. This is due to phosphorus compounds creating a coating that forms a carbon film on the foam surface to isolate oxygen and inhibiting combustion [37,38,39,40].

### 3.6. Limiting Oxygen Index (LOI) Analysis

**Figure 8:**
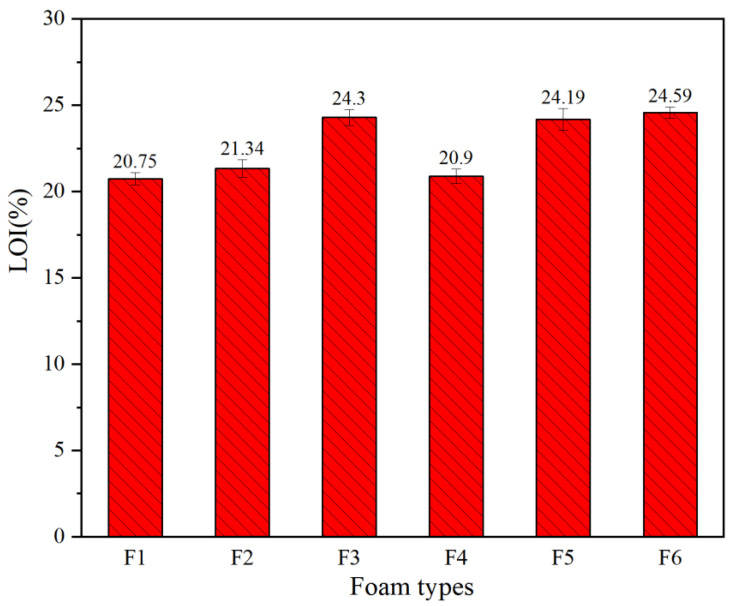
LOI of foams F1, F2, F3, F4, F5, and F6.

The flame retardancy of polymers can be effectively evaluated by the limiting oxygen index (LOI), which reflects the lowest oxygen concentration when the polymer burns in a mixed nitrogen atmosphere and oxygen [41]. The LOI test results for all foams are shown in Figure 8. Generally, a material is classified as flammable when its LOI value is below 22%, and when the LOI value is above 27%, the material can be classified as flame-retardant. As expected, the LOI values of foams F1, F2, and F4 were 20.75%, 21.34%, and 20.9%, respectively, indicating that these foams are flammable. Combined with the SEM images, it can be observed that the open cell size of the foams F1, F2, and F4 is not much different, resulting in a very small difference in the oxygen concentration inside the foams and thus similar LOI values. Therefore, they need the addition of a flame retardant if one wants to improve their flame retardancy. The foam F3 prepared using phosphoric acid presenting a good flame-retardant effect also presents an LOI value of 24.3%. The LOI values of foams F5 and F6 were 24.19 and 24.59, respectively. Thus, the proportion of glutaraldehyde added did not affect the LOI values much. The cause of this is that phosphate itself is commonly used as a flame retardant. The LOI test results of all foams also support the ignition test conclusion. Therefore, phosphoric acid as an initiator can improve the flame retardancy of GNIPU foam.

### 3.7. Thermogravimetric Analysis

**Figure 9:**
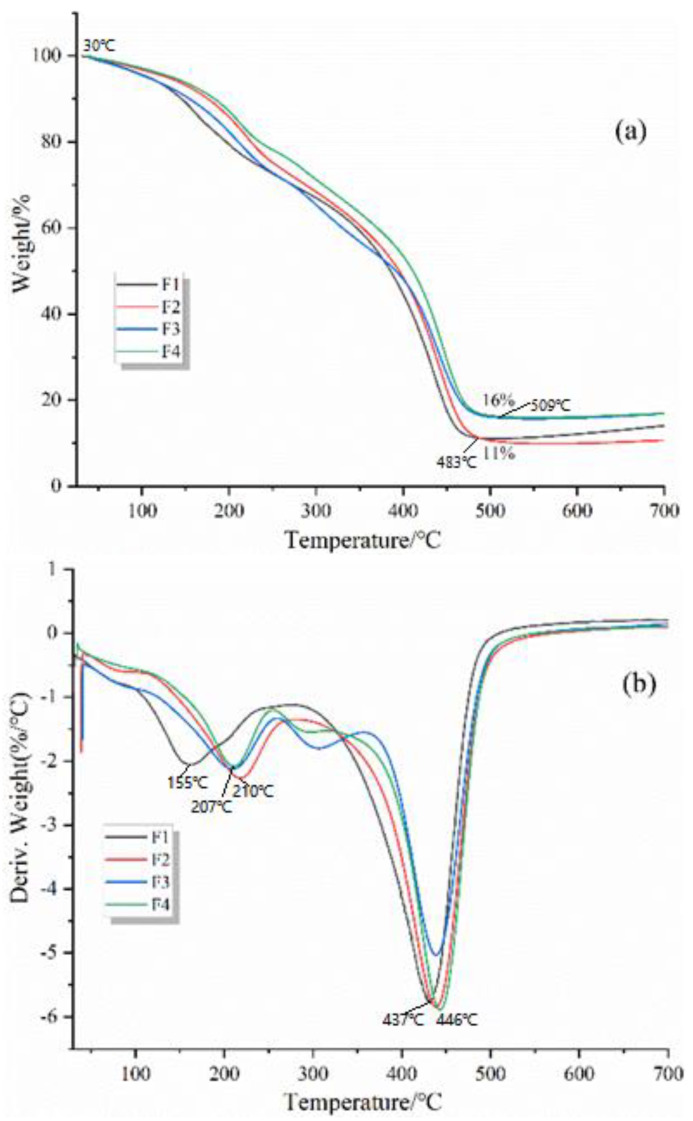
TG (**a**) and DTG (**b**) curves of GNIPU foams (F1, F2, F3, and F4).

Thermogravimetric analysis (TGA) was used to investigate the thermal stability of GNIPU foams. The TG (a) and DTG (b) curves of foams F1, F2, F3, and F4 under a N_2_ atmosphere are shown in Figure 9. It can be noticed that the pyrolysis behavior of all foams was mainly divided into two decomposition stages. The first mass loss stage occurs in the temperature range of 30–280 °C, which belongs to the elimination and evaporation of impurities and water adsorbed on the surface of the solid foam powder. In addition, some small-molecule carbohydrate impurities and unstable chemical bonds were decomposed at this stage [27,42]. At the same time, an excess of hexamethylenediamine also leads to a quality loss at this stage due to the boiling point of hexamethylenediamine being around 200 °C. The second mass loss stage occurs between 300 °C and 600 °C and is mainly a mass loss stage. In this stage, the foam cross-linked network is progressively degraded and eventually destroyed [6].

In Figure 9, some obvious differences in pyrolysis behavior can be observed when the foam was prepared with different acid catalysts. The final residual mass percentage at 700 °C shows that these foams have a different mass loss percentage, the residual mass of foam F1 and F2 being around 11%, and foam F3 and F4 have the least mass loss with about 16% residual mass. In the second mass loss stage, F3 loses significantly less mass than F4. Furthermore, the decomposition temperature of foam F3 moves to a higher temperature than the others. These phenomena indicate that phosphoric acid catalysis increased the thermal stability of GNIPU foams, which is consistent with the ignition test and LOI results.

### 3.8. Fourier Transform Infrared (FT-IR) Spectroscopy

**Figure 10:**
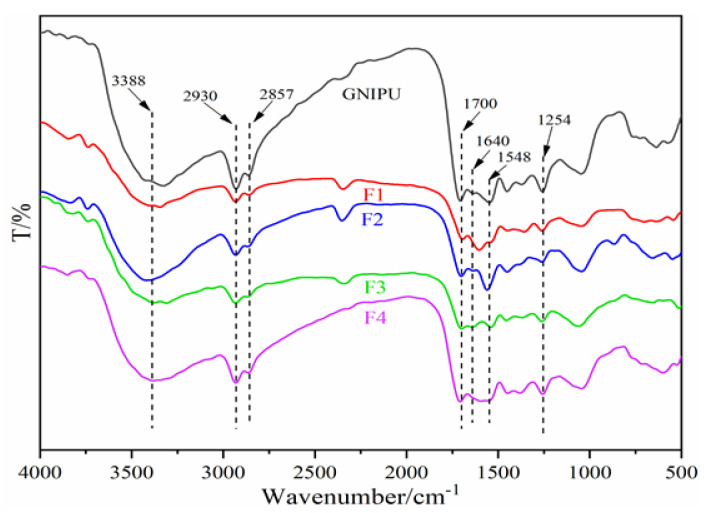
FT-IR spectra of GNIPU and foams (F1, F2, F3, and F4).

The Fourier transform infrared (FT-IR) spectra of the GNIPU and all foams are shown in Figure 10. In Figure 10, a wide absorption peak at 3388 cm^−1^ is attributed to the O-H and N-H stretching vibration. The absorption peaks at 2930 cm^−1^ and 2857 cm^−1^ are attributed to the –CH_2_– stretching vibration, which is related to the presence of HMDA [25,27]. Of these, the peaks at 1700 cm^−1^ and 1640 cm^−1^ are attributed to the C=O absorption of urethane groups and the N-H deformation of carbamate [43]. The urethane characteristic peak at 1548 cm^−1^ is related to C-N stretching [32,34]. In the FT-IR spectra of foams F1 and F4, the peaks at 1640 cm^−1^ are invisible due to the coincidence of the peaks of N-H and C-N. The absorption peak at 1254 cm^−1^ is characteristic of amino groups, which is decreased in F1, F2, F3, and F4 compared to GNIPU. This result is due to the acid and glutaraldehyde reaction with the amino groups in GNIPU.

### 3.9. SEM Analysis

**Figure 11:**
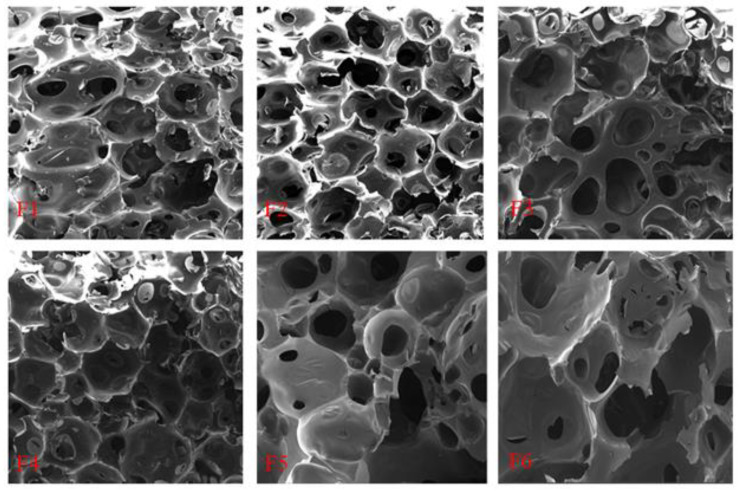
SEM pictures of GNIPU foams.

To explain the effects of the different catalysts on the F1, F2, F3, F4, F5, and F6 foam cell structures, these were observed by SEM and are shown in Figure 11. First, two different types of cells appear to be present in all foams, namely open and closed cells. It is easy to see that these foam structures mainly exist as open cells, which is caused by the evaporation of water in the resin during foaming and drying [6]. In addition, some rupture or debris, as well as incomplete cell structures, can be observed by SEM, this being due to the cutting process used to prepare the samples [41]. Second, by comparing different foams’ SEM images, it appears that foam F1 has more debris due to its more brittle cell walls. The foam F3 has the thickest cell walls, explaining the best compression resistance in Figure 3. It is worth noting that foam F4 appears to have a more complete cell morphology, which is attributed to the citric acid catalyst that can also help to strengthen and stabilize the three-dimensional structure of the foam by a reaction with its constituents [44,45].

**Figure 12:**
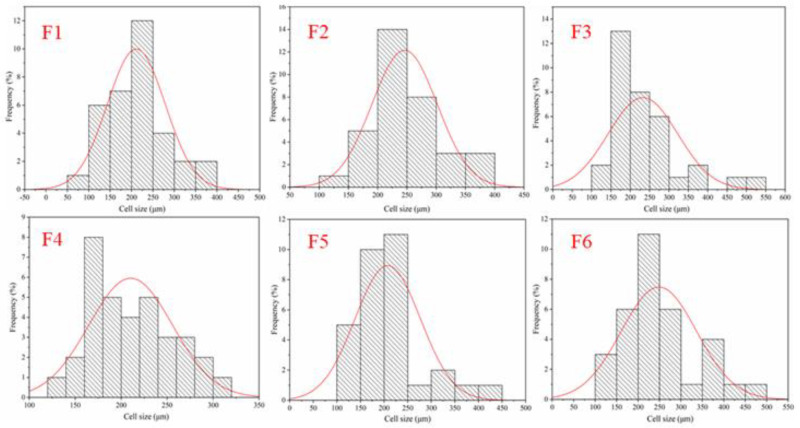
Cell size distributions of GNIPU foams. Gaussian cell size distribution curve indicated in red.

Figure 12 shows the results of the cell size distribution of GNIPU foams F1, F2, F3, F4, F5, and F6. It can be seen that the cell pore sizes of the four kinds of foam are mainly distributed in the 200–250 μm range. It can be noted that the cell pore size distribution of foam F4 prepared with citric acid is more concentrated. Furthermore, combining SEM pictures and Figure 12, the relationship of cell pore size is F2 > F6 > F3 > F5 > F4 > F1. In general, the foam pore size is inversely proportional to density. However, the cell pore sizes of the four foams are similar, also due to the fact that the density of the four foams is not much different.

## 4. Conclusions

In this paper, the effects of different acids and glutaraldehyde addition on foam morphology and properties were investigated. The six kinds of foams exhibited a similar foam morphology and cell pore size. The compression mechanical properties are different owing to the different acid catalysts used. Foam F3 prepared with phosphoric acid had better compressive strength above 65% compression. The ignition test and limiting oxygen index test results indicated that the foams F1, F2, and F4 show high flammability, belonging to inflammable materials. However, the foam prepared with phosphoric acid has a certain flame retardancy. Simultaneously, foam F3 also shows better thermal stability. Some of these low-cost and non-toxic self-blowing GNIPU foams have a wide application potential, in particular the one prepared with phosphoric acid as a catalyst.

## Figures and Tables

**Table 1:** Performance of the GNIPU foams.

Foam Number	Formulations	Density (g/cm^3^)	Ignition Time (s)
F1	10 g GNIPU + 1.0 g FA + 3.0 g G	0.119	75 s
F2	10 g GNIPU + 1.0 g MA + 3.0 g G	0.101	85 s
F3	10 g GNIPU + 1.0 g PA + 3.0 g G	0.105	0 s
F4	10 g GNIPU + 1.0 g CA + 3.0 g G	0.108	80 s
F5	10 g GNIPU + 1.0 g PA + 2.0 g G	0.107	0 s
F6	10 g GNIPU + 1.0 g PA + 4.0 g G	0.112	0 s

Note: FA—formic acid; MA—maleic acid; PA—phosphoric acid; CA—citric acid; G—glutaraldehyde.

## Data Availability

The original contributions presented in the study are included in the article, further inquiries can be directed to the corresponding authors.
